# Identification of a novel and plant height-independent QTL for coleoptile length in barley and validation of its effect using near isogenic lines

**DOI:** 10.1007/s00122-024-04561-9

**Published:** 2024-02-21

**Authors:** Shang Gao, Zhouyang Su, Jun Ma, Jian Ma, Chunji Liu, Huihui Li, Zhi Zheng

**Affiliations:** 1 CSIRO Agriculture and Food, 306 Carmody Road, St Lucia, QLD 4067 Australia; 2 State Key Laboratory of Crop Gene Resources and Breeding, Institute of Crop Sciences, Chinese Academy of Agricultural Sciences (CAAS), Beijing, 100081 China; 3 Nanfan Research Institute, Chinese Academy of Agricultural Sciences, Sanya, 572024 Hainan China; 4 Tasmanian Institute of Agriculture, University of Tasmania, Prospect, TAS 7205 Australia; 5 College of Agronomy and Biotechnology, China Agricultural University, Beijing, 100193 China; 6 State Key Laboratory of Crop Gene Exploration and Utilization in Southwest China, Triticeae Research Institute, Sichuan Agricultural University, Chengdu, China

## Abstract

**Key message:**

**This study reported the identification and validation of novel QTL conferring coleoptile length in barley and predicted candidate genes underlying the largest effect QTL based on orthologous analysis and comparison of the whole genome assemblies for both parental genotypes of the mapping population.**

**Abstract:**

Coleoptile length (CL) is one of the most important agronomic traits in cereal crops due to its direct influence on the optimal depth for seed sowing which facilitates better seedling establishment. Varieties with longer coleoptiles are preferred in drought-prone areas where less moisture maintains at the top layer of the soil. Compared to wheat, genetic study on coleoptile length is limited in barley. Here, we reported a study on detecting the genomic regions associated with CL in barley by assessing a population consisting of 201 recombinant inbred lines. Four putative QTL conferring CL were consistently identified on chromosomes 1H, 5H, 6H, and 7H in each of the trials conducted. Of these QTL, the two located on chromosomes 5H and 6H (designated as *Qcl.caf-5H* and *Qcl.caf-6H*) are likely novel and *Qcl.caf-5H* showed the most significant effect explaining up to 30.9% of phenotypic variance with a LOD value of 15.1. To further validate the effect of this putative QTL, five pairs of near isogenic lines (NILs) were then developed and assessed. Analysis of the NILs showed an average difference of 21.0% in CL between the two isolines. Notably, none of the other assessed morphological characteristics showed consistent differences between the two isolines for each pair of the NILs. Candidate genes underlying the *Qcl.caf-5H* locus were also predicted by employing orthologous analysis and comparing the genome assemblies for both parental genotypes of the mapping population in the present study. Taken together, these findings expand our understanding on genetic basis of CL and will be indicative for further gene cloning and functional analysis underly this locus in barley.

**Supplementary Information:**

The online version contains supplementary material available at 10.1007/s00122-024-04561-9.

## Introduction

Crop establishment is a crucial factor for achieving optimal yields and relies on rapid shoot emergence and leaf area development. The coleoptile, a protective structure found in Monocotyledons that is responsible for delivering the stem and first leaves from the embryo to the soil surface (Rebetzke et al. 2007a), plays an important role in the establishment. Longer coleoptiles are desirable, particularly in areas with low precipitation as they enable seeds to be sown at a deeper moisture level for better germination (Mahdi et al. 1998; Schillinger et al. 1998; Mohan et al. 2013). Numerous studies have shown positive correlations between coleoptile length (CL) and increased plant number with deep sowing (Schillinger et al. 1998; Takeda and Takahashi 1999; Matsui et al. 2002; Rebetzke et al. 2007b). In addition, longer coleoptiles are advantageous in conservation tillage practice, where crop residues are left on the soil surface. Cultivars with longer coleoptiles would also facilitate crop establishment when sowing into stubble, as the longer coleoptiles would be able to penetrate through the residue layer and reach the soil surface more easily which can minimize the potential negative effects on seedling emergence and establishment (Rebetzke et al. 2005). Furthermore, growers also benefit from deep seeding by avoiding animal threats (Brown et al. 2003) and pre-emergent herbicides damages (Brand et al. 2007).

It has been reported that plant height (PH) is associated with CL and semi-dwarf cultivars have relatively shorter coleoptiles than non-dwarf ones (Takeda and Takahashi 1999; Platz et al. 1999; Paynter and Clarke 2010). Two major dwarfing genes in wheat, *Rht1* and *Rht2*, have been associated with reductions in CL (Rebetzke et al. 2001; Ellis et al. 2002; Botwright et al. 2005;). Similarly, the semi-dwarfing gene *uzu* in barley has been found to inhibit the elongation of various tissues including reducing CL (Saisho et al. 2004; Chen et al. 2016). Additionally, many brachytic (*brh*) semidwarf mutants in barley also showed pleiotropic effects on CL (Dahleen et al. 2005). The widespread use of these dwarfing genes in modern cultivars as part of the Green Revolution has made it challenging to breed cultivars with increased CL and reduced PH. In contrast, gibberellin (GA)-sensitive dwarfing genes, such as *Rht8-14* in wheat and *sdw1* and *denso* in barley, have contributed to reducing PH without affecting CL (Zhang and Zhang 2003; Ellis et al. 2004; Botwright et al. 2005; Kuczyńska et al. 2013). These genes together with PH-independent QTL for CL enable the breeding of dwarf cultivars with long coleoptiles.

Previous studies have revealed significant variation for CL in barley (Grando and Ceccarelli 1995; Takeda and Takahashi 1999; Paynter and Clarke 2010) and investigating its genetic basis is essential for breeding programs. Despite the comprehensive studies on wheat, research on the mapping of quantitative trait loci (QTL) for CL remains to be limited in barley. So far, only four studies reported the identification of QTL conferring CL in barley. Takahashi et al. (2001) identified a QTL conferring CL on chromosome 5H by assessing two double haploid (DH) populations. Later, the same group reported the detection of six QTL that regulated CL on six chromosomes except 3H based on an analysis of a DH population of 150 lines (Takahashi et al. 2008). Ahmadi-Ochtapeh et al. (2015) detected three QTL associated with CL on chromosomes 2H, 4H and 6H under different levels of salt stress. More recently, Luo et al. (2020) conducted a genome-wide association study (GWAS) on a panel of 328 barley accessions and identified 12 intergenic loci associated with CL distributing on all seven chromosomes. Due to the low resolution provided by QTL mapping (Paterson et al. 1988), it is hard to conclude if any of these QTL were in the same chromosomal regions. Moreover, these QTL were only detected in a single mapping or natural population, and their effects in different genetic backgrounds are unknow. Thus, these loci must be treated as putative only.

Development of near isogenic lines (NILs) has been widely recognized one of the most effective approaches to minimize the interference of the genetic backgrounds when studying a targeted gene or locus. This is because NILs involve only two isolines in a particular genetic background allowing simplified assessment of the effect from a specific allele. Moreover, NILs have the advantage of fixing other morphological characteristics that may otherwise influence the accuracy of phenotyping (Pumphrey et al. 2007). These features enhance the utility of NILs in validating a wide range of characteristics of interest (Chen et al. 2012, 2016; Gao et al. 2019). There are two main methods for developing NILs, backcross introgression (Paterson et al. 1990; Dorweiler et al. 1993) and heterogeneous inbred family (HIF) analysis (Tuinstra et al. 1997). The former method only provides a single genetic background and can be labor intensive and time consuming. In contrast, the later approach is more preferred due to its ability to generate NILs with a diverse range of recombinant genetic backgrounds. By incorporating different genetic backgrounds, the effect of a specific QTL on the observed phenotype can be more accurately assessed and disentangled from other genetic and environmental factors (Tuinstra et al. 1997).

The study reported here attempted to identify novel QTL conferring CL in barley. To achieve this, we assessed a population consisting of 201 recombinant inbred lines (RILs) previously generated from a study by Zhou et al. (2021) and generated NILs targeting the most stable QTL to further validate its effect. Candidate genes underlying this QTL were also predicted by utilizing the available whole genome assemblies of both parents and sequence homology analysis. Results obtained from these investigations are presented in this publication.

## Materials and methods

### Plant materials

A population of 201 RILs, derived from a cross between Morex and AWCS276 (Zhou et al. 2021), was assessed in this study. Morex is a six-row malting barley with spring habit (Rasmusson and Wilcoxson 1979), and AWCS276 is a two-row wild barley with winter habit. This population was advanced to F9 generation using single-seed descendent method and fast generation procedures (Zheng et al. 2013, 2023).

### Phenotypic evaluation

Four trials (designated as *RIL01*, *RIL02*, *RIL03*, and *RIL04*) were carried out by assessing the RIL population and parental lines in a controlled environment facility (CEF) at Queensland Bioscience Precinct (QBP). Each trial consisted of three replicates with 14 seedlings per genotype in each replicate. High-quality seeds without any visible damages were collected from mainstems of each genotype from the glasshouses. Seeds were treated with 70% ethanol for 30 s and then washed twice with distilled water. Sterilized seeds were germinated in Petri dishes on two layers of filter paper saturated with water. To ensure uniform germination, the Petri dishes were placed in a cold room at 4 °C for two days. Subsequently, the dishes were transferred to room temperature and kept overnight before planting into the potting mix. The seedlings with approximately 0.5 cm length were sown in square punnets of a 56-well tray (Rite Grow Kwik Pots, Garden City Plastics, Australia). The trays were filled with steam-sterilized University of California (UC) mix composed of 50% sand and 50% peat (v/v) and arranged in a randomized block design in the darkened CEF, where a constant temperature of 15 °C was maintained (Spielmeyer et al. 2007). The CLs of the etiolated seedlings were measured after 14 days using a ruler as the distance from the scutellum to the tip of the coleoptile. The average values of 14 seedlings for each genotype were used for the further analyses.

### QTL analysis

A previously published genetic map which incorporated 1140 polymorphic markers derived from genotyping by sequencing (GBS) data for this population was adopted in this study (Zhou et al. 2021). The linkage map encompassed a total length of approximately 1022.4 cM, with an average distance of 0.7 cM. QTL analysis was performed using MapQTL 6.0 software with interval mapping function (Ooijen and Kyazma 2009). For each CL trial, a test of 1000 permutations was performed to identify the LOD threshold corresponding to a genome-wide false discovery rate of 5% (*P* < 0.05). Based on the permutation test, a threshold LOD value was used to determine the significance of a QTL. To visually represent the positions of the identified QTL, linkage maps were generated using MapChart (Voorrips 2002). Confident interval was defined for each QTL as the 1.5-LOD drop off on either side of the peak marker (Visscher et al. 1996). The flanking markers for each QTL were determined by identifying the nearest markers that bordered the support interval.

### Development and assessment of near isogenic lines targeting the largest effect QTL identified

Development of NILs was carried out using the HIF method described by Tuinstra et al. (1997). A marker (*CL-5H*) closely linked with the peak of the largest effect QTL was generated and used to develop NILs (Table S1). PCR reactions for the marker were conducted using 12 μL mixtures consisting of 20 ng genomic DNA, 0.2 μmol L^−1^ of forward and reverse primer, 2.4 mmol L^−1^ MgCl_2_, 1 mmol L^−1^ dNTPs, and 0.5 U *Taq* DNA polymerase. The PCR reaction procedure was as followed: one cycle of 5 min at 94 °C, 35 cycles of 1 min 94 °C, 1 min at annealing temperature 60 °C and 1 min at 72 °C, with a final extension of 10 min at 72 °C. The PCR products were visualized and separated on 3.0% agarose gels mixed with Gel-Red stain. Development of NILs was as described by Gao et al. (2019). Briefly, the closely linked marker was used to identify heterozygous plants from *F*5 lines of the RIL population. The identified heterozygous plants were self-pollinated, and eight to ten plants derived from each of the heterozygous plants were used for the next round of selection. This process was repeated until the *F*8 generation. From each heterozygous individual, two plants were chosen based on the marker profile of *CL-5H*, one carrying the allele from Morex (*M*) and other carrying the allele from AWCS276 (*A*). These two selected lines were considered as a putative NIL pair for further phenotypic assessment.

These putative NILs obtained were evaluated in three independent trials in the CEF at QBP. Phenotypic assessments of CL were conducted as described above. To investigate the differences between two isolines of each NIL pair in terms of other agronomic traits, including plant height (PH), heading date (HD), kernel length (KL), kernel width (KW), spike row type (SRT), kernel number per spike (KNPS), fertile tiller number (FTN) and thousand kernel weight (TKW), three trials were conducted in glasshouses at QBP. Each glasshouse trial comprised three replicates, with each replicate consisting of five plants for each of the isolines. These plants were sown in a separate 2.0 L pot filled with steam-sterilized UC mix. A random block design was used for all the trials. The glasshouse conditions were set as follows: a photoperiod of 16 h, day/night temperature of 25/18 (± 5) °C, and day/night relative humidity of 65/80 (± 5)%. Measurements of PH, HD, KL, KW, KNPS and TKW were conducted using the main tillers from each replicate, following the protocol outlines in a previous study (Zheng et al. 2022). SRT was determined by categorizing the spikes as either two or six-row types. FTN were calculated from each of the five plants in each replicate. The average values of five plants from each replicate were used for further analysis of the agronomic traits.

### Statistical analysis

The best linear unbiased prediction (BLUP) for different trials and the broad-sense heritability (*H*^*2*^) were calculated using the PROC MIXED and VARCOMP procedures of SAS V8.0 (SAS Institute, Cary, NC, USA; https://www.sas.com). To estimate random effects, the BLUP for the phenotypic values were calculated according to the model: *Y*_i_ = *X*_i_*f* + *a*_*i*_ + *e*_*i*_, where *f* = *a* vector of fixed effects, *X*_i_ = an incidence vector, *e*_i_ = the environmental deviation, and *a*_*i*_ = the phenotypic value (Goddard 1992)*.* The overall *H*^2^ for CL was estimated as *H*^2^ = *σ*^2^_g_/(*σ*^2^_g_ + *σ*^2^_ge_/*n* + *σ*^2^_e_/*nr*), where *σ*^2^_g_ is the genetic variance, *σ*^2^_ge_ is the *G* × *E* variance, *σ*^2^_e_ is the error, *n* is the number of environments, and *r* is the number of replicates. Normal distribution test, Student’s *t-*test (*P* < *0.05*) and correlation analysis of phenotype values in different trials were analyzed by SPSS18.0 software (SPSS, Chicago, IL, USA).

We used the random intercepts model to analysis the differences of phenotypic values between the two (*M*- and *A*-) isolines of the five NIL pairs. This was performed using the *R* (R Core Team 2020) package lme4 (Bates et al. 2015). For the ${i}^{{\text{th}}}$ subject at the ${j}^{{\text{th}}}$ measurement occasion:

$$Y_{ij} = X_{i} \beta + Z_{j} + \varepsilon_{ij}$$

where ${Y}_{ij}$ is the response variable, $\beta$ is the vector of regression parameters, ${X}_{i}$ is the fixed effect, ${Z}_{j}$ is the random effect, and ${\varepsilon }_{ij}$ is a random noise following the Gaussian distribution. We processed the data for each of the five NIL pairs separately and treated replicates of the experiment as the random effect. *M*-isolines were set as the experimental group, and *A*-isolines were set as the control group. In particular, we set the fixed effect of *A*-isolines to 0 and that of *M*-isolines was considered as 1. Thus, by the construction of the random intercepts model, we can directly obtain the average value difference between the two groups through $\beta$.

### Prediction of candidate genes underlying QTL for coleoptile length

The process of identifying candidate genes in the targeted region was conducted as described by Zheng et al. (2022). Initially, physical positions of the largest effect QTL were determined by blasting the sequences of the flanking makers against barley Morex V3 pseudomolecules (Mascher et al. 2021). Coding sequences and protein sequences of the genes within the targeted region were obtained from https://wheat.pw.usda.gov/GG3/content/morex-v3-files-2021 for Morex and NCGR wild barley database https://www.ncgr.ac.cn/wild_barley for AWCS276 (Liu et al. 2020). Only the genes obtained sequence variations between the two parents were considered as potential candidates. Gene sequences related to CL, cell elongation, auxins response factors and GA synthesis pathway were also collected from rice and blasted against Morex. SNP variations of the candidate genes and their functional annotations were identified using Snippy v4.3.6 with default settings (https://github.com/tseemann/snippy), while DIAMOND v2.0 was used to detect reciprocal best hits for analysis of the protein sequences (Buchfink et al. 2021).

## Results

### Characterization of coleoptile length in the RIL population

**Fig. 1:**
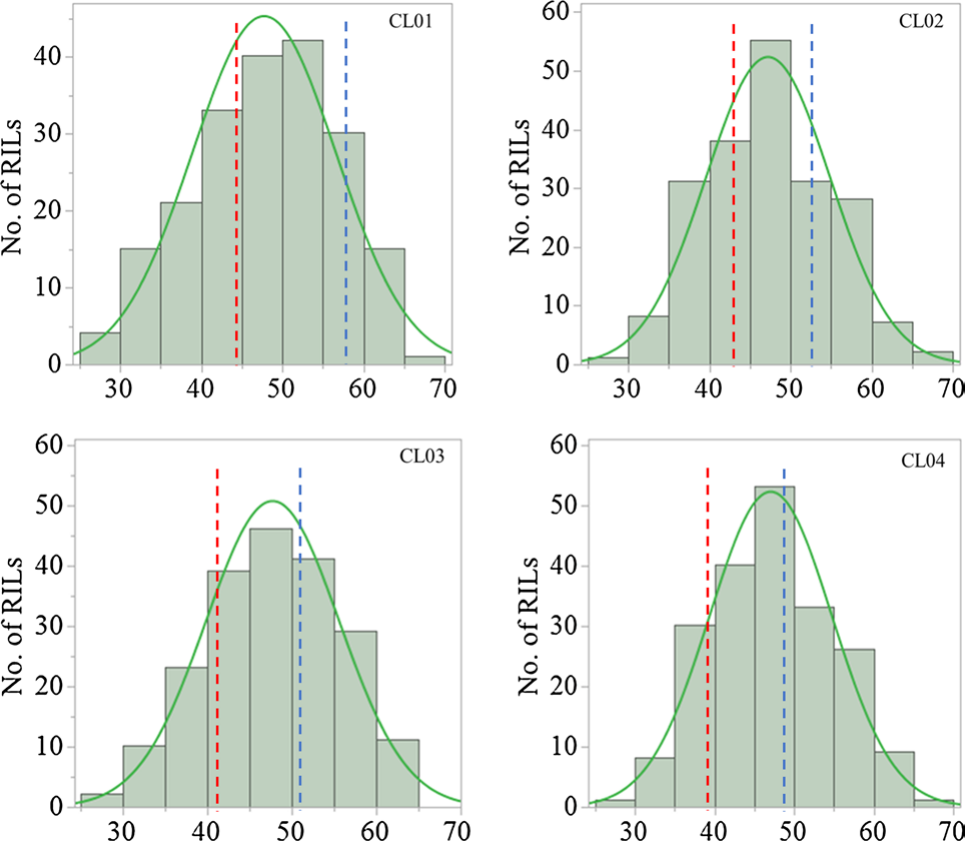
Frequency distributions for coleoptile length obtained from the population of Morex/AWCS276 in different trials. Vertical lines indicate average coleoptile length of parents: Morex (red) and AWCS276 (blue) (Colour figure online)

CL values were significantly and positively correlated among four different trials and the BLUP dataset (*P* < *0.01*), with correlation coefficients ranging from 0.74 to 0.95 (Table S2). The genotype AWCS276 had consistently greater CL than Morex in all four trials conducted. The CL of the mapping population ranged from 25.7 to 67.4 mm and the standard deviation (SD) ranged from 7.7 to 8.8 mm (Table 1). The broad-sense heritability (*H*^*2*^) was estimated to be 0.94, suggesting that the variation in CL is predominantly influenced by genetic factors (Table 1). In addition, the frequency distribution of CL values indicated continuous variation with transgressive segregation (Fig. 1).

**Table 1:** Phenotypic variation and heritability of coleoptile length for the parents and population assessed in different environments

Trait	Trials	Parents		Population					
		Morex	AWCS276	Min	Max	Mean	SD	CV(%)	H^2^
CL (mm)	*RIL01*	44.8	57.9	25.7	67.4	47.7	8.8	18.5	0.94
	*RIL02*	42.6	52.2	28.8	65.4	47.2	7.6	16.2	
	*RIL03*	40.8	50.2	27.5	63.3	47.7	7.9	16.6	
	*RIL04*	39.5	48.9	29.1	65.2	47.1	7.7	16.3	

*CL* coleoptile length, *SD* standard deviation, *CV* coefficient of variation, *H*^*2*^ the broad-sense heritability

### QTL for coleoptile length

**Fig. 2:**
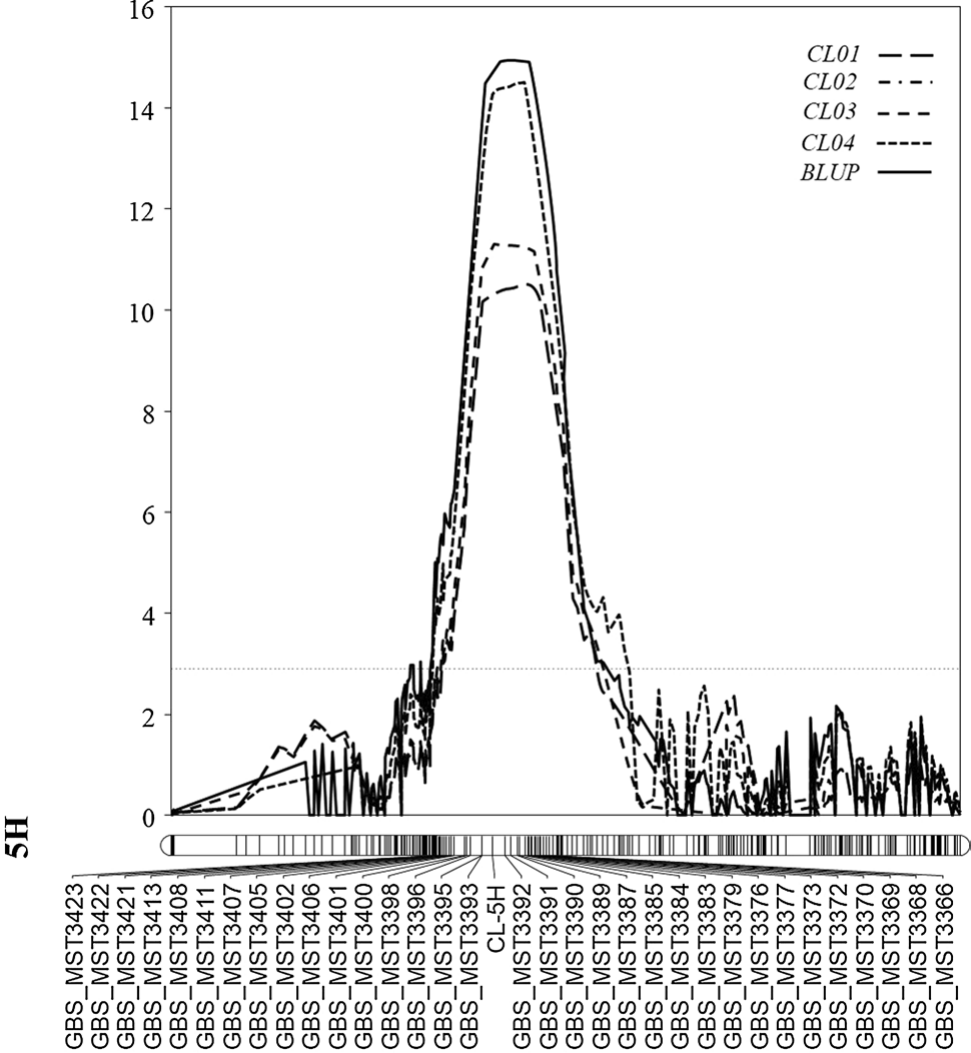
QTL conferring coleoptile length detected on chromosome 5H with interval mapping from the population of Morex/AWCS276. The LOD values from each centimorgan of the chromosome were plotted against the chromosome, and the vertical dotted line indicates the average significant threshold (LOD = 2.9) derived from permutation test. CL-5H indicates the marker developed close to the peak of this QTL. The short arm of the chromosome is on the left side while the long arm is on the right side

Based on the results from permutation tests, a total of five putative QTL associated with CL were identified on chromosomes 1H, 4H, 5H, 6H, and 7H with the LOD values exceeding the threshold of 2.9 (Table 2). Among these five putative QTL, four were consistently expressed in each of the four trials as well as with the use of the BLUP dataset. Notably, the QTL located on chromosome 5H, designated as *Qcl.caf-5H*, showed the largest effect explaining up to 30.9% of phenotypic variance with a maximum LOD value of 15.1 (Table 2; Fig. 2). The allele for long coleoptile of the *Qcl.caf-5H* was derived from the parent AWCS276. Based on the flanking markers identified from the support interval, the *Qcl.caf-5H* interval covered a physical distance of 27.5 Mbp from 435.2 to 459.7 Mbp.

**Table 2:** QTL for coleoptile length identified in the population of Morex/AWCS276^#^

Trials	Chr	QTL	Peak (cM)	Linkage map interval (cM)	Physical map interval (Mbp)	Left marker	Right marker	LOD	PVE (%)	Add
*RIL01*	1H	*Qcl.caf-1H*	107.9	103.9–114.8	493.3–506.6	GBS_MST624	GBS_MST632	3.8	8.4	0.26
	5H	*Qcl.caf-5H*	70.2	66.6–80.5	436.2–459.7	GBS_MST3400	GBS_MST3392	10.2	20.8	− 0.41
	6H	*Qcl.caf-6H*	48.4	44.6–57.0	240.3–299.7	GBS_MST4431	GBS_MST4088	5.9	12.5	0.32
	7H	*Qcl.caf-7H*	110.6	104.1–112.7	587.5–596.8	GBS_MST4657	GBS_MST4617	3.5	7.6	0.24
*RIL02*	1H	*Qcl.caf-1H*	109.3	103.9–114.8	493.3–506.6	GBS_MST621	GBS_MST633	3.9	8.6	0.23
	4H	*Qcl.caf-4H*	59.5	51.3–61.2	476.8–497.6	GBS_MST2888	GBS_MST2970	3.3	7.3	0.22
	5H	*Qcl.caf-5H*	74.3	63.6–77.8	435.3–459.5	GBS_MST3402	GBS_MST3387	14.0	28.8	− 0.41
	6H	*Qcl.caf-6H*	50.5	44.6–57.0	240.3–299.7	GBS_MST4431	GBS_MST4088	5.1	11.1	0.26
	7H	*Qcl.caf-7H*	108.9	104.1–112.7	587.5–596.8	GBS_MST4657	GBS_MST4617	4.1	8.9	0.23
*RIL03*	1H	*Qcl.caf-1H*	109.0	103.9–114.8	493.3–506.6	GBS_MST624	GBS_MST632	4.5	9.9	0.25
	5H	*Qcl.caf-5H*	71.2	62.5–80.3	430.2–459.7	GBS_MST3407	GBS_MST3372	11.3	23.8	− 0.39
	6H	*Qcl.caf-6H*	51.5	44.6–57.0	240.3–299.7	GBS_MST4431	GBS_MST4088	5.7	12.2	0.28
	7H	*Qcl.caf-7H*	110.7	104.1–112.7	587.5–596.8	GBS_MST4657	GBS_MST4617	3.7	8.2	0.22
*RIL04*	1H	*Qcl.caf-1H*	109.5	103.9–114.8	493.3–506.6	GBS_MST624	GBS_MST632	4.5	9.9	0.23
	5H	*Qcl.caf-5H*	68.7	66.6–74.3	435.2–459.7	GBS_MST3400	GBS_MST3390	14.6	29.8	− 0.42
	6H	*Qcl.caf-6H*	50.4	44.6–57.0	240.3–299.7	GBS_MST4431	GBS_MST4088	5.6	11.9	0.26
	7H	*Qcl.caf-7H*	108.9	104.1–112.7	587.5–596.8	GBS_MST4657	GBS_MST4617	3.5	7.6	0.23
*BLUP*	1H	*Qcl.caf-1H*	108.4	103.9–114.8	493.3–506.6	GBS_MST621	GBS_MST633	6.8	14.7	0.27
	4H	*Qcl.caf-4H*	58.3	55.9–61.2	476.8–492.6	GBS_MST2946	GBS_MST2970	3.5	7.7	0.25
	5H	*Qcl.caf-5H*	71.2	66.6–80.4	436.2–459.7	GBS_MST3408	GBS_MST3392	15.1	30.9	− 0.50
	6H	*Qcl.caf-6H*	50.3	44.6–57.0	240.3–299.7	GBS_MST4431	GBS_MST4088	5.9	12.5	0.30
	7H	*Qcl.caf-7H*	107.5	104.1–112.7	587.5–596.8	GBS_MST4657	GBS_MST4617	4.4	9.5	0.23

*Chr* chromosome, *Peak* position of the QTL peak, *BLUP* best linear unbiased prediction, *cM* centimorgan, *PVE* phenotype variance explained, *Add* Additive effect (positive values: alleles for long coleoptile from Morex, negative values: alleles for long coleoptile from AWCS276)

Three additional QTL were also stably expressed across all four trials and the BLUP dataset. They were detected on chromosomes 1H (*Qcl.caf-1H*), 6H (*Qcl.caf-6H*) and 7H (*Qcl.caf-7H*) and explained phenotypic variances ranging from 7.6 to 14.7% with corresponding LOD values between 3.5 and 6.8 (Table 2). The fifth QTL was located on chromosome 4H (*Qcl.caf-4H*); however, this QTL was only detected in one of the four trials and BLUP dataset explaining up to 7.8% with a maximum LOD value of 3.5 (Table 2). The alleles for long coleoptile of these four QTL were all derived from Morex.

### Development and assessment of NILs targeting the Qcl.caf-5H locus

**Fig. 3:**
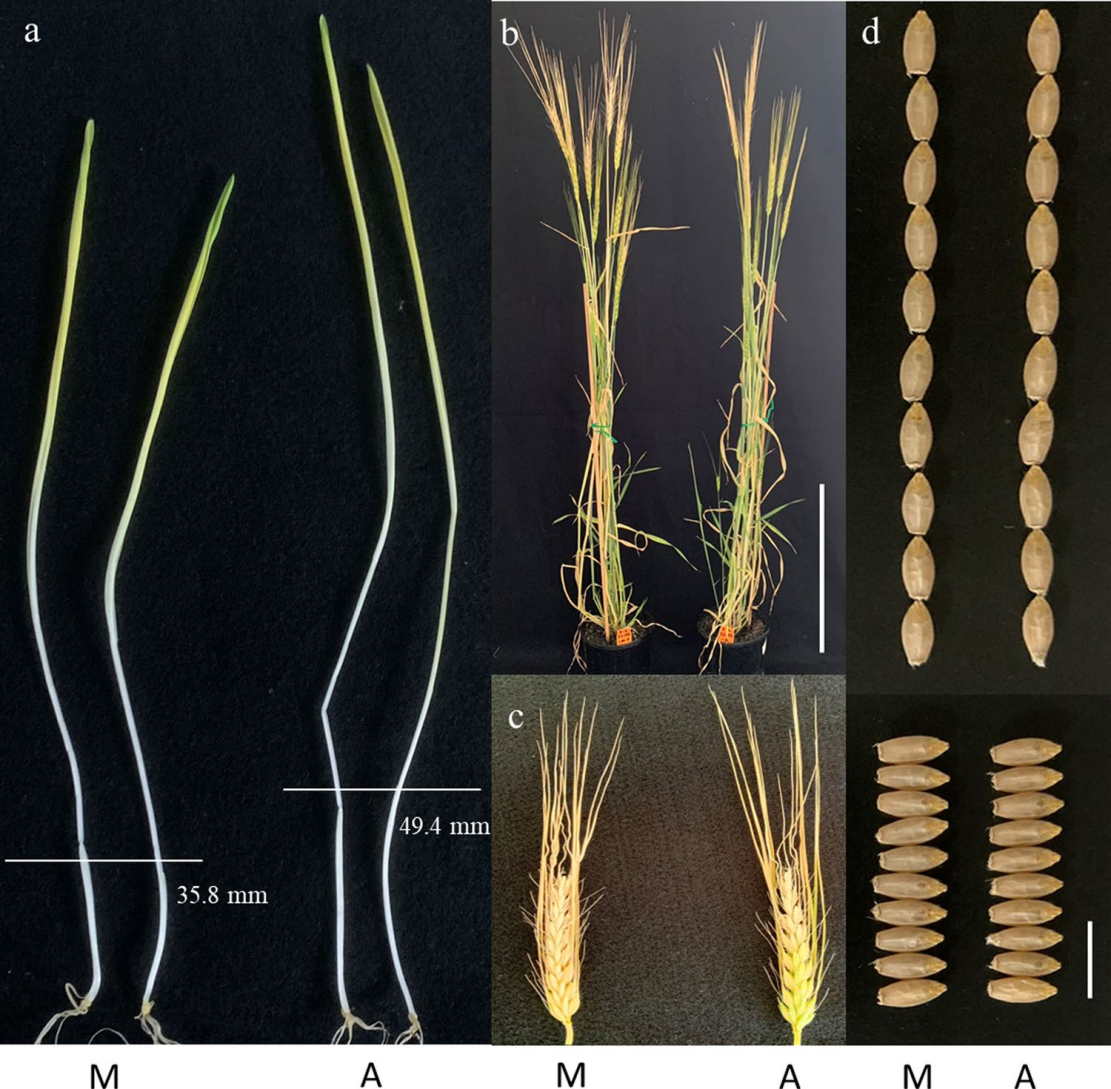
Morphologies of a pair of the near isogenic lines (NIL1) showing **a** the difference in coleoptile length; **b** similar plant height and flowering time. Scale bar is 40 cm; **c** similar spike morphology, and **d** similar seed morphology. Scale bar is 1 cm. *M* represents lines with the Morex allele and *A* represents lines with the AWCS276 allele

As *Qcl.caf-5H* was consistently detected with the most significant effect, NILs targeting this locus were obtained to further validate its effect and detect its relationship with other agronomic traits. Of the eight putative NIL pairs initially generated based on the marker profiles of *CL-5H* (Table S1), five showed significant differences in CL between the two isolines ranging from 14.5% to 28.2% with an average of 21.0% (Table 3). The crossovers between marker *CL-5H* and *Qcl.caf-5H* are assumed to be the reason for three of the eight NILs pairs were not statistically different for CL. As expected, differences for any of the other characteristics assessed (including PH, HD, KL, KW, KNPS, SRT, FTN and TKW) were not consistently detected between the two isolines for any of the five NIL pairs (Table 3; Fig. 3).

**Table 3:** Difference in various characteristics measured between the two isolines for each of the five pairs of near isogenic lines targeting the coleoptile length locus on chromosome 5H estimated by a random intercept model

Isoline	CL (mm)	PH (cm)	HD	SRT	FTN	KNPS	TKW (g)	KW (mm)	KL (mm)
NIL_1_AWCS276	46.50	91.77	3	2	15.44	16.11	42.73	3.00	8.99
NIL_1_Morex	35.60	90.88	3	2	15.66	15.56	42.60	2.99	8.81
Mean Diff. (%)	23.44	0.98	0	0	− 1.95	3.11	0.23	0.33	2.00
Diff. Estimate	− 1.09***	− 0.88	0	0	0.22	− 0.56	− 0.13	− 0.01	− 0.19**
NIL_2_AWCS276	63.62	114.73	3	2	20.0	61.33	45.13	3.16	10.20
NIL_2_Morex	52.26	113.49	3	2	20.0	60.67	45.19	3.07	10.23
Mean Diff. (%)	17.92	1.05	0	0	0.00	0.98	− 0.22	2.85	− 0.29
Diff. Estimate	− 1.14***	− 1.24	0	0	0.00	− 0.67	0.06	− 0.09	0.02
NIL_3_AWCS276	56.78	114.61	2	6	17.67	20.56	34.01	3.04	9.65
NIL_3_Morex	40.82	113.61	2	6	17.33	19.33	33.99	3.01	9.65
Mean Diff. (%)	28.17	0.87	0	0	2.26	6.31	0.29	0.99	0.00
Diff. Estimate	− 1.59***	− 1.00	0	0	− 0.33	− 1.22	− 0.02	− 0.03	0.00
NIL_4_AWCS276	47.65	115.77	1	6	23.22	62.94	33.57	3.04	10.13
NIL_4_Morex	37.76	116.39	1	6	22.11	62.31	33.57	3.03	10.13
Mean Diff. (%)	20.75	− 0.52	0	0	4.74	0.95	0.00	0.33	0.00
Diff. Estimate	− 0.99***	0.61	0	0	− 1.11	− 0.63	0.00	− 0.01	0.00
NIL_5_AWCS276	40.63	86.56	2	6	22.11	17.67	45.88	3.04	9.50
NIL_5_Morex	34.70	87.33	2	6	21.44	17.11	45.61	3.02	9.56
Mean Diff. (%)	14.53	− 0.81	0	0	3.17	3.39	0.65	0.66	− 0.63
Diff. Estimate	− 0.59***	0.78	0	0	− 0.67	− 0.56	− 0.27	− 0.01	0.06

*AWCS276* *and* *Morex* allele from AWCS276 and Morex, *Diff.* difference, *CL* coleoptile length, *PH* plant height, *HD* heading date, *SRT* spike row type, *FTN* fertile tiller number, *KNPS* kernel number per spike, *TKW* thousand kernel weight, *KL* kernel length, *KW* kernel width

** and *** indicate a statistically significant difference at the probability levels of 0.01 and 0.001, respectively

### Prediction of candidate genes underlying the Qcl.caf-5H locus

A total of 253 genes were detected within the *Qcl.caf-5H* region and 103 of them obtained non-synonymous substitutions between the two parental genotypes resulting in changes in protein functions (Table S3). To further narrow down the list of candidate genes, sequences for 356 genes related to CL, cell elongation, auxins response factors and GA synthesis pathway were obtained from rice for an orthologous analysis (Table S4). Screening these genes against Morex detected three orthologs (including *HORVU.MOREX.r3.5HG0481610*, *HORVU.MOREX.r3.5HG0482670* and *HORVU.MOREX.r3.5HG0486380*) in the *Qcl.caf-5H* interval. Sequence similarities of these genes between rice and barley ranged from 67.8 to 92.3% (Table 4). Seven SNPs were identified in exons of *HORVU.MOREX.r3.5HG0481610* between the two parents, and two of them resulted in non-synonymous mutations at positions 694 (Tyrosine → Aspartic acid) and 1077 (Lysine → Asparagine) (Table 4), leading to amino acid residue substitutions. Four SNPs were detected for *HORVU.MOREX.r3.5HG0486380* when comparing the two parental genotypes. Three of these SNPs were non-synonymous mutations (G → A transition at position 299, T → C transition at position 535 and T → G transversion at position 1050, respectively) producing amino acid residue substitutions at positions 100 (Aspartic acid → Glycine), 179 (Proline → Serine) and 350 (Leucine → Phenylalanine), respectively (Table 4). Additionally, there were nine SNPs between Morex and AWCS276 in exons of *HORVU.MOREX.r3.5HG0482670*; however, they were all synonymous mutations (Table 4).

**Table 4:** Candidate genes and their orthologs underlying the locus conferring coleoptile length on chromosome 5H

Barley orthologs	Physical position (bp)	Rice gene	Identity	SNP^#^	Amino acids^#^
HORVU.MOREX.r3.5HG0481610	Chr5H:437130805–437145874	*OsYUC9*	71.6	C/G(486)	Y/D(232)
				G/C(540)	K/N(359)
				C/T(572)	
				T/G(694)	
				C/A(957)	
				T/C(975)	
				A/C(1077)	
HORVU.MOREX.r3.5HG0482670	Chr5H:441915631- 441920070	*OsCesA9*	92.3	A/C(1449)	None
				T/C(1470)	
				C/T(1620)	
				T/C(2211)	
				G/A(2223)	
				C/G(2253)	
				C/T(2475)	
				C/A(2502)	
				G/A(3075)	
HORVU.MOREX.r3.5HG0486380	Chr5H:459445692-459449302	*OsbZIP72*	67.8	G/A(299)	D/G(100)
				T/C(535)	P/S(179)
				T/A(1002)	L/F(350)
				T/G(1050)	

^#^ the numbers in brackets represent the positions of differences in nucleotide or amino acid sequences between Morex and AWCS276 relative to initiation codons

## Discussion

CL is a major determinant of increased early vigor and better crop establishment as well as deep sowing tolerance. Short coleoptiles pose challenges in many cereals often leading to issues such as poor establishment, late emergence, and slow early leaf area development particularly in water-limited growing regions. Previous studies also indicated that strong correlations between CL and drought resistance index (Wang and Zou 1997; Hu et al. 2007) and suggested that CL can be used as a surrogate trait to screen for drought tolerant genotypes at seedling stage (Khadka et al. 2020; Wei et al. 2022). Thus, identification of genomic regions contributing to CL is highly valuable to breeding programs. In this study, four stable QTL were detected in barley and the one namely *Qcl.caf-5H* had the largest effect explaining up to 30.9% of phenotypic variance. NILs targeting this locus were developed and assessed to validate its effect, showing that the isolines containing this QTL would increase the CL by an average of 21.0%. Importantly, the *Qcl.caf-5H* locus does not affect other agronomic traits which would facilitate the breeding of barley varieties with improved CL. Moreover, with availability of the high-quality genome assemblies for both parents, candidate genes in the *Qcl.caf-5H* locus interval were predicted and two of them containing non-synonymous mutations would form the primary targets for further gene cloning.

It is of note that although previous studies reported several loci controlling CL (Takahashi et al. 2008; Ahmadi-Ochtapeh et al. 2105; Luo et al. 2020), none of them seem to overlap with the loci on the chromosomes 5H and 6H reported here. Three putative QTL conferring CL have been previously reported on the chromosome 5H at approximate positions of 12.3 Mbp, 382.1 Mbp and 582.4 Mbp, respectively (Takahashi et al. 2001, 2008; Luo et al. 2020). However, they were at least 50 Mbp away from the *Qcl.caf-5H* locus (peak at 442.3 Mbp within the internal range of 435.2–459.7 Mbp) detected in the present study, suggesting this locus is a novel one for CL. Moreover, the *Qcl.caf-6H* is likely another novel QTL located near the centromere on the chromosome 6H within the interval between 232.6 and 277.2 Mbp. This is in contrast to the QTL reported on the short arm of this chromosome (Takahashi et al. 2008; Ahmadi-Ochtapeh et al. 2105) and the one reported by Luo et al. (2020) located at the distal end of the long arm on chromosome 6H. Additionally, the other two consistent QTL on the chromosomes 1H and 7H were located at the regions similar to those identified from previous studies (Takahashi et al. 2001, 2008). However, further investigation is required to determine if they are the same ones as QTL mapping only provides limited resolution (Paterson et al. 1988).

The present study used a single marker closely linked to the peak of the QTL instead of flanking markers (Pumphrey et al. 2007) for the development of NILs. While the use of flanking markers can minimize the risk of failure, it can lead to linkage drag and large fragment if closely linked markers with the targeted locus are not available (Zhou et al. 2021). Using one marker closely linked with the targeted locus allowed to obtain NILs with minimized sizes of undesired chromosomal segments that differ between the two isolines. However, it is of note that this approach has the risk of generating false NILs because potential recombination events may occur between the linked marker and the targeted QTL (Ma et al. 2012; Gao et al. 2019). This is likely the reason why three of the putative NIL pairs developed from this study did not find significant differences in CL between the two isolines, which is similar to the findings from previous studies (Ma et al. 2012; Gao et al. 2019; Zhou et al. 2021). Moreover, considering the limited resolution given by the QTL mapping, the marker derived from such studies may not be reliably used in tagging the targeted loci (Paterson et al. 1988). Therefore, further investigation is required for the development of markers co-segregating with the *Qcl.caf-5H* locus, which would facilitate the identification of the prevalence of this locus in various barley accessions.

NILs are valuable resources for validating the effects at a given locus as well as for exploring potential relationships with other agronomic traits. Analyzing the NILs targeting the *Qcl.caf-5H* locus in the present study did not only validate its effects but also showed this CL locus is not associated with PH. It has been reported that PH can impact CL in genotypes carrying the GA-insensitive dwarf genes including *Rht1*, *Rht2*, *uzu* and *brh* (Rebetzke et al. 2001; Ellis et al. 2002; Botwright et al. 2005; Chen et al. 2016). However, given the preference for shorter varieties with longer coleoptiles, it is crucial to identify QTL conferring CL that are independent of PH for breeding programs. Therefore, the identification of the *Qcl.caf-5H* locus as PH-independent would facilitate the breeding of long coleoptile barley varieties with reduced PH, particularly when combined with GA-sensitive semi-dwarf genes which have no significant correlations with CL (Zhang and Zhang 2003; Kuczyńska et al. 2013). Moreover, the NILs would have significant potentials for studying the effects of CL on maintaining plant number and achieving high grain yields through different sowing depths under field conditions (Rebetzke et al. 2007b). Such investigations can provide valuable insights into crop management strategies and the potential for improved yields in practical agricultural settings.

As high-quality genome assemblies are available for both parents of the mapping population used in this study, such comprehensive genomic information enhanced our ability to identify and predict candidate genes within a specific genomic region (Zhou et al. 2021; Zheng et al. 2022). Any gene within the *Qcl.caf-5H* region that showed sequence differences between the two parents especially those containing non-synonymous variations (Table S3) can be considered a candidate. Comparisons of the sequences between Morex and AWCS276 for genes with known effects on CL in rice enabled us to further shorten the list of candidate genes. Subsequently, three genes emerge as primary targets within this interval. One of the genes, *HORVU.MOREX.r3.5HG0481610* is orthologous to *OsYUC9* in rice, which is involved in auxin biosynthesis (Khanday et al. 2023; Kim et al. 2023). Auxins can modify plant cell walls and are crucial for coleoptile cell elongation and expansion (Philippar et al. 1999; Catalá et al. 2000). The second one, *HORVU.MOREX.r3.5HG0486380*, is homologous with *OsbZIP72* in rice*.* It has been reported that overexpression of this gene can enhance submerged seed germination and coleoptile elongation by directly binding to the promoter of alcohol dehydrogenase (Wang et al. 2021). The third gene, *HORVU.MOREX.r3.5HG0482670* is orthologous to *OsCesA9* in rice, which is involved in secondary cell wall cellulose synthesis and plant growth (Tanaka et al. 2003). Mutants of *OsCesA9* have been demonstrated to have reduced cellulose crystallinity leading to thinner secondary cell walls (Li et al. 2017). Although this gene only obtains synonymous variations between the two parental genotypes, it cannot be ruled out due to the factors that synonymous variants have the potentials to alter mRNA structures which may affect translation rates or efficiency (Hunt et al. 2014) as well as protein production and abundance (Komar 2016). Thus, further investigation of this gene is warranted to determine its potential functional significance in relation to CL regulation.

### Supplementary Information

Below is the link to the electronic supplementary material.

Supplementary file1 (DOCX 16 KB)

Supplementary file2 (XLSX 1218 KB)

## Data Availability

The datasets generated during and/or analyzed during the current study are available from the corresponding author upon reasonable request.
