# Supplementary material for: Identification of a novel and plant height-independent QTL for coleoptile length in barley and validation of its effect using near isogenic lines
Source: Theor Appl Genet. 2024 Feb 21;137(3):53. doi: 10.1007/s00122-024-04561-9 (PMC10881613; doi:10.1007/s00122-024-04561-9)
Supplement: Supplementary file 1 — Supplementary file1 (DOCX 16 KB) [file 122_2024_4561_MOESM1_ESM.docx]

**Table S1.** Primers for the Indel maker developed closely linked wiht the peak of the *Qcl.caf-5H* based on sequence difference between two parental genotypes

| Primer | Primer sequence | Product size (bp) |
| --- | --- | --- |
| CL-5H | Forward - CGAGCGAGTCAAGTGAGGTG | 228 |
|  | Reverse - TTCGCCGGGAAACATCATCA |  |

**Table S2.** Correlation coefficients of coleoptile length in the population of Morex/AWCS276 among the four trials and BLUP dataset^#^

| Trial | *RIL01* | *RIL02* | *RIL03* | *RIL04* | *BLUP* |
| --- | --- | --- | --- | --- | --- |
| *RIL01* | 1.00 |  |  |  |  |
| *RIL02* | 0.91** | 1.000 |  |  |  |
| *RIL03* | 0.93** | 0.95** | 1.000 |  |  |
| *RIL04* | 0.89** | 0.94** | 0.94** | 1.000 |  |
| *BLUP* | 0.74** | 0.91** | 0.79** | 0.93** | 1.00 |

^#^*RIL01, RIL02, RIL03 and RIL04* four independent trials conducted for assessing coleoptile length, *BLUP* best linear unbiased prediction, **significant at *P* < 0.01.
